# Replacement of oxytocin bolus administration by infusion: influences on postpartum outcome

**DOI:** 10.1007/s00404-015-3916-6

**Published:** 2015-11-04

**Authors:** Julia J. Löytved-Hardegg, Mirjam Brunner, Jean-Jacques Ries, Stefanie von Felten, Christina Heugel, Olav Lapaire, Cora Voekt, Irene Hösli

**Affiliations:** Department of Obstetrics, University Hospital of Basel, Spitalstrasse 21, 4031 Basel, Switzerland; Cantonal Hospital of Aarau, Women’s Hospital, Tellstrasse, 5001 Aarau, Switzerland; Clinical Trial Unit, University Hospital of Basel, Schanzenstrasse 55, 4031 Basel, Switzerland; Hospital of Grabs, Women’s Hospital, Spitalstrasse 44, 9472 Grabs, Switzerland

**Keywords:** Oxytocin, Post partum haemorrhage, Third stage of labour

## Abstract

**Purpose:**

Postpartum haemorrhage (PPH) represents a leading cause of maternal morbidity and mortality. Giving oxytocin after birth reduces the risk for PPH. It has never been tested whether different methods of oxytocin administration affect the maternal outcome. This study aims to compare the infusion versus the bolus application of oxytocin after singleton vaginal delivery.

**Methods:**

This retrospective monocentre study compares the incidence of clinically relevant postpartum complications in women receiving 5 IE of oxytocin as a bolus or as a 100 ml-infusion over 5 min, given immediately after birth. Included were women delivering singletons vaginally at term. We used propensity score weighting to compare outcomes between women receiving bolus and infusion and to minimize the selection bias in this retrospective cohort.

**Results:**

1765 patients were included. Patient characteristics were balanced. We found no significant differences for the combined overall postpartum adverse outcome (the incidence of PPH, manual removal of the placenta and/or curettage). For the single outcomes, we observed a significantly higher frequency of manual removal of the placenta (Odds ratio 1.47, 95 % CI 1.02–2.13) and a slightly higher but clinically not relevant estimated blood loss (Relative effect 1.05, 95 % CI 1.01–1.10) in the infusion group.

**Conclusion:**

The data show a tendency towards more complications in the infusion group. It is related to a more frequent need for manual removal of the placenta.

## Introduction

Postpartum haemorrhage (PPH) represents a leading cause of maternal morbidity and mortality [30] and the administration of oxytocin immediately after birth has been shown to reduce the risk for PPH [4, 20].

Along with the vasoconstrictive effect on the uterine vessels, oxytocin results in peripheral vasodilatation, hypotension and tachycardia, causing in rare cases even myocardial ischemia with significant ST-segment depression [8, 14]. Maternal deaths due to cardiac arrest after the administration of oxytocin in women with unstable cardiac function have been reported [28]. In order to decrease the risk of oxytocin-related cardiovascular side effects, administration of oxytocin as a bolus has been replaced in Switzerland, as well as other countries, by a short infusion over 5 min [16]. This has been the method of administration of the obstetrics department of the University Hospital Basel since December 2010. It remains unclear, however, whether oxytocin infusion is equally effective as the bolus for preventing PPH and associated adverse events after vaginal delivery.

Epidemiological studies have shown a trend towards an increasing prevalence of PPH since 1995 due to different reasons [3, 13]. To our knowledge it has never been tested whether the administration modalities contribute to this trend.

This retrospective cohort study aims to compare the incidence of postpartum adverse outcome in women receiving an infusion of oxytocin and women receiving a bolus of oxytocin directly after vaginal delivery.

## Methods

### Participants

We conducted a retrospective, monocentre analysis in women who delivered vaginally at the University Hospital of Basel from January 2010 to August 2011. The data set were retrieved from an electronic database and patient medical records. Exclusion criteria were multiple gestation, caesarean delivery, preterm delivery <36 weeks of gestation and stillbirth.

Women were classified into two groups, a historical group (bolus group, before December 2010) and a current group (infusion group, since December 2010). In the historical group women received an intravenous bolus of 5 International Units (IU) of oxytocin directly after the birth of the baby. In the current group an infusion of 5 IU oxytocin in 100 ml NaCl 0.9 % over 5 min immediately after delivery was applied.

### Clinical outcome measures

The primary outcome was the incidence of at least one postpartum adverse outcome (PPH, manual removal of the placenta and/or curettage). Secondary outcomes were the incidence of each component of the primary outcome, as well as the incidence of severe PPH, placenta retention >30 min, uterine atony, red blood cell transfusion, transfer to the intensive care unit (ICU), estimated blood loss (ml), decrease in serum haemoglobin (g/l) and duration of the third stage of labour (min).

PPH was determined as an estimated blood loss >500 ml and severe PPH as >1000 ml within 24 h after delivery, as estimated by the obstetrician. Placenta retention was defined as a placenta that had not undergone expulsion within 30 min after delivery. Haemoglobin levels were recorded ante partum and within 72 h postpartum to determine the decrease in haemoglobin levels resulting from the birth.

### Sample size estimation

The baseline rate of postpartum adverse outcomes was estimated from a pilot subgroup of 320 patients, consisting of 160 women in each group. A postpartum adverse outcome was observed in 18.1 % of patients in the infusion group versus 12.5 % in the bolus group. Based on these results, a sample size of *n* = 1740 was determined for our study (870 per group), using a χ^2^ test and aiming at a statistical power of 90 % at a significance level, α, of 5 %.

### Data analysis

A total of 1765 patients, of whom 892 received oxytocin as a bolus and 873 as an infusion, were finally included in the data analysis. The total number of deliveries during the study period was 3705.

At first we performed naïve comparisons of the primary and secondary outcomes, determining the frequency of categorical outcomes and the median, mean and standard deviation for the continuous outcomes among the study arms (bolus vs. infusion). To get “naive effect size estimates” we used logistic regression models with “infusion” (1 = infusion, 0 = bolus) as an explanatory factor for the categorical outcome variables and standard linear regression models for the continuous outcome variables. All continuous outcomes were log-transformed to meet the assumption of normal errors.

To account for potential confounding with other variables, which must be expected due to the observational nature of the retrospective data we then performed a propensity score weighted analysis. A propensity score was estimated for each patient as the probability to have received an infusion (as opposed to a bolus), based on the patient characteristics shown in Tables 1 and 2. We included as potential confounders all known and available risk factors as well as patient characteristics possibly influencing the outcomes tested in this investigation. Propensity scores (*p*) were estimated by generalized boosted logistic regression. This method iteratively minimizes the imbalance of covariates between two groups (infusion vs. bolus) [25]. Imbalance was defined as the average effect size difference across all covariates. We used inverse probability weighting (IPW) to estimate the effect of the treatment on outcome variables [12]. Patients in the infusion group received weight 1/*p* (i.e., small propensity scores *p* resulted in large weights) and patients in the bolus group received weight 1/(1 − *p*) (i.e., large *p* resulted in large weights).

**Table 1:** Patient characteristics and potential risk factors for PPH in the two study groups bolus and infusion

		Bolus group (*n* = 892)	Infusion group (*n* = 873)	*P*
Gravidity^a^				0.006
	Multigravida	550 (61.7 %)	481 (55.1 %)	
	Primigravida	342 (38.3 %)	392 (44.9 %)	
Parity^a^				0.00095
	Multipara	448 (50.2 %)	369 (42.3 %)	
	Primipara	444 (49.8 %)	504 (57.7 %)	
Operative vaginal delivery		208 (23.3 %)	242 (27.7 %)	0.04
Induction of labour		184 (20.6 %)	173 (19.8 %)	0.72
Oxytocin for labour augmentation		564 (63.2 %)	579 (66.3 %)	0.19
Epidural anaesthesia		451 (50.6 %)	463 (53.0 %)	0.32
Severe perineal tears		77 (8.6 %)	72 (8.2 %)	0.84
Fetal macrosomia (>95th percentile)		17 (1.9 %)	17 (1.9 %)	1.00
Preeclampsia		8 (0.9 %)	10 (1.1 %)	0.78
Gestational diabetes		27 (3.0 %)	36 (4.1 %)	0.27
Other systemic disease		173 (19.4 %)	148 (16.9 %)	0.20
Hospitalization before 34 weeks of pregnancy^b^		3 (0.3 %)	13 (1.5 %)	0.02
Infertility treatment		33 (3.7 %)	30 (3.4 %)	0.87
p PPH		21 (2.4 %)	22 (2.5 %)	0.94
p Caesarean section		57 (6.4 %)	42 (4.8 %)	0.18
p Curettage		159 (17.8 %)	148 (16.9 %)	0.67
p Manual removal of the placenta		18 (2.0 %)	19 (2.2 %)	0.95
p Myomectomie		6 (0.7 %)	7 (0.8 %)	0.97
Maternal age		30.95 (±5.29)	30.86 (±5.35)	0.77
BMI at birth		28.40 (±4.65)	28.08 (±4.35)	0.19
Duration of labour (h)		11.69 (±10.25)	12.99 (±12.12)	0.0084
Duration of labour after rupture of membranes (h)		7.70 (±11.39)	8.63 (±13.40)	0.29
Gestational age at birth (days)		279.60 (±7.48)	279.52 (±7.79)	0.85
Newborn’s weight (g)		3451.32 (±450.32)	3423.79 (±423.35)	0.25

Frequencies (percentages) and *P* values from a *χ* ^*2*^ test are shown for categorical variables. The mean (±standard deviation) and *P* values from a Mann–Whitney test are shown for continuous variables. *n* number, *p* previous

^a^Gravidity and parity are shown as binary variables. However, actual gravidity and parity were used as continuous variables for propensity score estimation

^b^Because of impending preterm labour

**Table 2:** Descriptive analysis of the primary and secondary outcomes in the two study groups bolus and infusion

	Bolus group (*n* = 892)	Infusion group (*n* = 873)	*P*
Overall postpartum adverse outcome	138 (15.5 %)	142 (16.3 %)	0.70
PPH	131 (14.7 %)	133 (15.2 %)	0.80
Severe PPH	29 (3.2 %)	34 (3.9 %)	0.55
Manual removal of the placenta	28 (3.1 %)	42 (4.8 %)	0.09
Curettage	40 (4.5 %)	45 (5.2 %)	0.58
Placenta retention >30 min	57 (6.4 %)	61 (7.0 %)	0.68
Uterine atony	30 (3.4 %)	32 (3.7 %)	0.83
Red blood cell transfusion	7 (0.8 %)	13 (1.5 %)	0.24
Transfer to the ICU	10 (1.1 %)	8 (0.9 %)	0.85
Estimated blood loss (ml)	439.96 (±332.91)	463.17 (±323.83)	0.00019
Decrease in haemoglobin (g/l)	17.24 (±15.11)	18.12 (±14.73)	0.12
Duration of the third stage of labour (min)	11.88 (±12.32)	12.34 (±12.46)	0.17

Frequencies (percentages) and *P* values from a *χ* ^2^ test are shown for categorical outcomes. The mean (±standard deviation) and *P* values from a Mann–Whitney test are shown for continuous outcomes

A separate, propensity score”-weighted logistic regression model with infusion as explanatory factor was fitted for each categorical outcome variable. A standard linear regression model was used in case of the log-transformed continuous outcome variables.

All data analyses and figures were performed using the statistical software R (version 3.0.2) [22], using the package twang for propensity score estimation and inverse probability weighting [23].

## Results

### Patient characteristics

A comparison of the patient characteristics for the two study groups is shown in Table 1. Patient characteristics were already quite balanced before propensity score weighting, which was apparent from the few significant differences between the study groups. There were more primiparous and fewer multiparous women in the infusion group. Duration of labour was on average 1.3 h longer in the infusion group. Furthermore, there were more hospitalizations due to impending preterm labour during pregnancy and more operative vaginal deliveries in the infusion group.

The two groups did not significantly differ in risk factors leading to PPH such as previous PPH or previous manual removal of the placenta, previous caesarean section, previous curettage or previous myomectomy. The rates of infertility treatment, induction of labour, maternal diseases such as gestational diabetes or preeclampsia, epidural anesthesia, severe perineal tears and foetal macrosomia were all comparable in both groups.

**Fig. 1:**
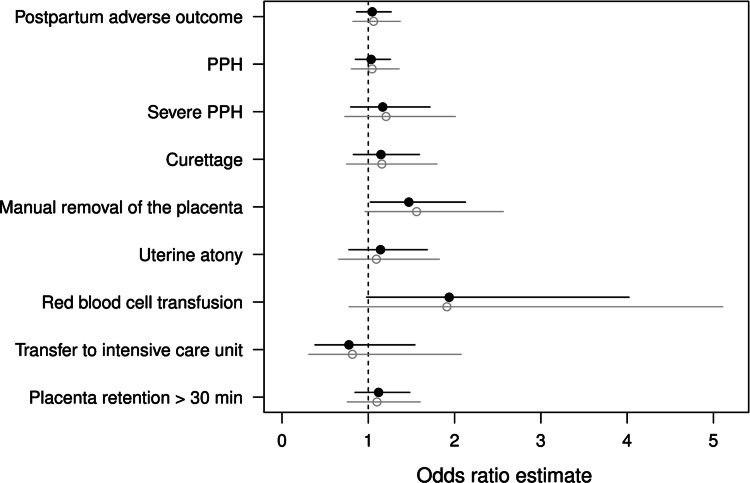
Propensity score-based (*black*) and naïve (*grey*) odds ratio estimates and 95 % confidence intervals for the effect of the oxytocin infusion versus bolus for all categorical outcomes

**Fig. 2:**
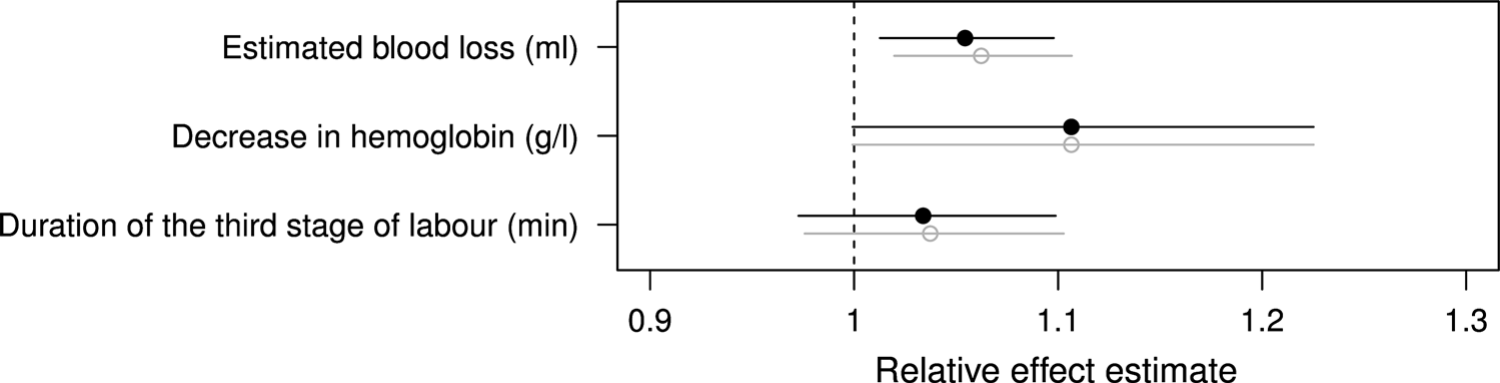
Propensity score based (*black*) and naïve (*grey*) estimates and 95 % confidence intervals for the relative effect of the oxytocin infusion versus bolus for the continuous outcomes

Figures 1 and 2 show the naïve and propensity score based odds ratio estimates and relative effect estimates for the primary and secondary outcomes. Table 2 shows the descriptive and Tables 3 and 4 the propensity score based data analysis. Reporting the results of our study we focus on the propensity score based odds ratio estimates (OR) and relative effects (RE).

**Table 3:** Propensity score-based odds ratio estimates (OR) and 95 % confidence intervals (CI) for the effect of infusion versus bolus for all categorical outcomes

	OR (95 % CI)	Pr (>∣z∣)
Postpartum adverse outcome	1.05 (0.86–1.26)	0.647
PPH	1.03 (0.85–1.26)	0.742
Severe PPH	1.17 (0.80–1.71)	0.428
Curettage	1.15 (0.83–1.59)	0.412
Manual removal of the placenta	1.47 (1.02–2.13)	0.038
Uterine atony	1.14 (0.78–1.68)	0.501
Red blood cell transfusion	1.94 (0.98–4.02)	0.063
Transfer to intensive care unit	0.78 (0.38–1.54)	0.470
Placenta retention >30 min	1.12 (0.85–1.48)	0.429

**Table 4:** Propensity score-based estimates and 95 % confidence intervals (CI) for the relative effect (RE) of infusion versus bolus for the continuous outcomes

	RE (95 % CI)	Pr (> ∣z∣)
Estimated blood loss (ml)	1.05 (1.01–1.10)	0.010
Decrease in haemoglobin (g/l)	1.11 (1.00–1.23)	0.052
Duration of the third stage of labour (min)	1.03 (0.97–1.10)	0.284

### Primary outcome

We observed similar frequencies of the primary outcome parameter, postpartum adverse outcome, in the two study groups (Table 2, top row).

Accordingly, we found no significant differences between the two groups with regard to the primary outcome, neither in the naïve nor in the propensity score based analyses (top of Fig. 1, Table 3).

### Secondary outcomes

We observed a significantly higher frequency of manual removal of the placenta in the infusion group than in the bolus group (OR 1.47, 95 % CI 1.02–2.13) (Fig. 1; Table 3). Moreover, the estimated blood loss was significantly higher in the infusion group than in the bolus group (RE 1.05, 95 % CI 1.01–1.10) (Fig. 2; Tables 2, 4). No significant differences between the groups were found for all other secondary outcomes (Figs. 1, 2; Tables 2, 3 and 4).

There appeared to be a trend towards a larger decrease in haemoglobin and a higher frequency of red blood cell transfusion in the infusion group although this did not reach statistical significance (decrease in haemoglobin: RE 1.11, 95 % CI 1.00–1.23, red blood cell transfusion: OR 1.94, 95 % CI 0.98–4.02) (Figs. 1, 2; Tables 3, 4).

Altogether, almost all odds ratios and relative effects suggested a less favourable outcome in the infusion group compared to the bolus group, albeit mostly without statistical significance.

## Discussion

Postpartum administration of oxytocin is known to decrease the risk of PPH (by at least 50 %) [4, 18, 20, 29]. Historically it was common to use an intravenous or intramuscular bolus of 5–10 IU oxytocin for the prevention of PPH. Several regimens of postpartum oxytocin for preventing PPH have been studied under varying conditions [1, 7, 17, 24, 26, 31]. At our institution, the application of 5 IU of oxytocin after the delivery of the baby is routine. So far, we are not aware of any study comparing the effect of 5 IU of oxytocin given as a bolus or as a short infusion on postpartum adverse outcome.

In this retrospective observational study, the rate of postpartum adverse outcomes was as high as 15.5 % in the bolus versus 16.3 % in the infusion group. Thus, the incidence of the three major postpartum adverse outcomes (primary outcome: PPH, curettage or manual removal of the placenta) did not change with the administration of oxytocin as a short infusion. Considering the secondary outcomes, however, there was a higher frequency of manual removal of the placenta and a slightly higher, but not clinically relevant, estimated blood loss after the oxytocin infusion compared to the bolus administration, both reaching statistical significance.

It is known that oxytocin is released in a pulsatile rhythm, increasing its pulse frequency during labour and reaching maximum frequency in the second stage of labour [10]. Accordingly, it was reported from clinical trials that a pulsatile administration of oxytocin is more effective in inducing labour than a continuous infusion [32]. Continuous infusion of oxytocin is thought to cause receptor desensitization [2, 19], and the haemodynamic effects of a second dose of 5 IU are attenuated compared to those seen after the first dose [14].

Considering the pharmacokinetic characteristics of oxytocin its application in the third stage of labour, during which myometrial oxytocin receptors may already exhibit desensitization, the higher efficiency of the oxytocin bolus could be explained by the higher plasma concentration flooding the receptors. Given the rather long time of about 40 min to reach a steady state and its short half-life, it seems possible that the plasma concentration achieved by the oxytocin infusion is not high enough to cause the same effect on uterine contractions as the bolus application.

The incidence of PPH and placenta retention in our study were higher than reported in systematic reviews, which demonstrated rates of 2–3 % for placenta retention and 9 % for PPH in developed countries [6, 15]. Our institution serves as a tertiary centre for high-risk pregnancies, and, therefore, conditions leading to PPH might occur more frequently here compared to the centres involved in the other studies.

To our knowledge there is so far no randomized controlled trial investigating the effects of the mode of application of postpartal oxytocin on vaginal deliveries.

The present investigation has several intrinsic limitations due to its retrospective study design. To adjust for the intrinsic bias by confounding resulting of the retrospective study design we choose a propensity score weighted analysis. Although there were statistically significant differences between the study groups concerning the parity, the duration of labour and the frequency of hospitalizations due to impending preterm labour during pregnancy, the comparability of the groups has been ensured best possible by propensity score-weighted data analysis. Data about the antepartum administration of oxytocin for labour augmentation, the induction of labour by prostaglandins and/or oxytocin and the duration of labour were incorporated into the calculation of propensity scores, as oxytocin exposure for labour augmentation is supposed to be an independent risk factor for PPH [9, 11]. Nevertheless, we had no data about the length and intensity of antepartum oxytocin administration in our patients. It is known that continuous exposure to oxytocin causes a desensitization of the human myometrium cells [19]. It has been shown that the dose requirements to achieve satisfactory uterine contractions are much higher in women having caesarean section for labour arrest (including oxytocin augmentation) than in those undergoing elective caesarean delivery [2, 5]. These differences in the responsiveness of oxytocin receptors probably also influence the efficiency of oxytocin in the third stage of labour given as bolus or infusion. However, during the study period there was no change in the time management of the first or second stage of labour.

A further limitation of this study consists in the rate of the oxytocin infusion: it was defined as 5 IU in 100 ml over 5 min; however, this was not administered by intravenous pump and, therefore, was not given at equal rates in all cases.

The peripartal blood loss, estimated by the attending obstetrician at a time, was significantly higher in patients receiving the oxytocin infusion—(even though this mean difference of about 23 ml was without clinical significance). Blood loss estimation by the obstetrician—in particular by varying obstetricians—however, is known not to be a very precise method. The finding of a (almost significant) trend towards a larger decrease of haemoglobin within 72 h postpartum and a higher frequency of red blood cell transfusions in the infusion group is supporting the impression of a higher blood loss in the infusion group, even though in further investigations the blood loss should be measured, not estimated. Pursche et al. made a similar observation when they compared the pre- and postpartum haemoglobin in all women who had undergone caesarean section in 2011 in the University Hospital of Schleswig–Holstein, Campus Luebeck: they found a significantly higher blood loss in those patients treated with an postpartum oxytocin infusion compared to those treated with an oxytocin bolus [21].

Although our study focused on the obstetrical adverse outcomes, a further limitation consists in the lack of information concerning maternal side effects.

Severe maternal side effects of oxytocin are dangerous but rare events [27, 28], especially in proportion to the rates of placental retention or severe PPH, which may result in much more serious adverse outcomes. The risk of severe cardiovascular side effects caused by oxytocin should be weighed up with the risk of PPH and associated complications.

In conclusion, the data show a tendency towards an increased incidence of adverse maternal outcome after postpartum oxytocin infusion compared to bolus administration of oxytocin. The mode of administration did not affect the primary outcome, but was associated with a more frequent need for manual removal of the placenta and higher peripartum blood loss. The substantial limit of this study consists in its retrospective study design. Nevertheless, its results show a trend of clinical importance that should be reviewed in a randomized controlled trial.
